# Few-Shot Cross-Bridge Damage Diagnosis from Vibration Sensor Signals via Siamese Contrastive Pretraining with Self-Calibrated Convolution

**DOI:** 10.3390/s26134153

**Published:** 2026-07-01

**Authors:** Zixu Hu, Wei He, Haitao Li, Yongweng Wu

**Affiliations:** School of Civil Engineering, Anhui University of Science and Technology, Huainan 232000, China

**Keywords:** vibration sensors, structural health monitoring, sensor signal processing, cross-bridge transfer learning, few-shot damage diagnosis, siamese contrastive pretraining

## Abstract

Vibration sensor networks deployed on bridges continuously generate large volumes of unlabelled measurements under healthy operation, whereas labelled damage records on any specific target bridge remain extremely scarce—a chronic data asymmetry that constrains data-driven structural health monitoring (SHM). Existing remedies either require labelled source-bridge data or borrow augmentation pipelines and encoders from computer vision that are poorly matched to one-dimensional vibration signals. This study proposes a two-stage framework—siamese contrastive pretraining followed by few-shot fine-tuning on the target bridge—that learns environment-invariant representations from unlabelled source-side sensor signals and transfers them to a new bridge using only a handful of labelled samples. Three contributions are advanced: (i) a signal-domain augmentation policy that decouples sensor-level corruptions from operational-level fluctuations, including a frequency-band stochastic masking scheme designed to emulate cross-bridge perturbations; (ii) a one-dimensional self-calibrated convolutional encoder embedded in a stop-gradient siamese learner, providing the enlarged receptive field and inter-channel coupling required to capture sparse damage signatures in multi-sensor recordings; and (iii) a transferability analysis that formally links the contrastive invariance objective to a bound on the expected cross-bridge risk. On the Z24 benchmark and an in-house four-configuration laboratory bridge population, the method attains a 5-shot macro-F1 of 0.913 (Z24 → Lab) and 0.892 (Lab → Z24), outperforming eleven baselines by 3.4–37.1 percentage points.

## 1. Introduction

### 1.1. Background and Motivation

Bridges constitute load-bearing arteries of modern transportation networks, and their progressive deterioration under coupled actions of fatigue, corrosion, environmental cycling, and increasingly frequent climate extremes makes timely damage diagnosis a matter of public safety as much as of asset management [1,2]. Over the past three decades, structural health monitoring (SHM) has matured from a research curiosity into a routinely deployed engineering practice, with on-bridge sensor arrays continuously streaming acceleration, strain, displacement, and environmental observations [3]. The corresponding data analytics, propelled by deep neural architectures, have demonstrated remarkable success in identifying damage signatures from long-term response records, with particular advances in convolutional and recurrent encoders for one-dimensional vibration signals [4,5]. Recent work has further coupled convolutional and recurrent units with channel-attention mechanisms to handle environmental confounders; for instance, Huang et al. reconstructed structural acceleration responses using a CNN-BiGRU network with squeeze-and-excitation attention while explicitly compensating for environmental temperature effects [6]. Such studies underscore that environmental variability is a primary obstacle to reliable data-driven SHM—precisely the obstacle our cross-bridge invariance objective is designed to overcome.

Notwithstanding these advances, real-world deployment is held back by a stubborn data asymmetry. Historical ambient response from a healthy bridge can be accumulated for years and is genuinely abundant; in stark contrast, labelled response records covering distinct damage states are intrinsically rare, because deliberately damaging an in-service bridge for the sake of dataset construction is neither economically tolerable nor ethically acceptable [7]. The asymmetry intensifies whenever the monitored bridge is recently built or has never undergone a documented damaging event. As Quqa et al. observed in their regional-scale SHM survey, individual bridges within a transportation network rarely possess the diversity of labelled examples required to train supervised classifiers, and the cost of instrumenting every structure for the duration needed to accumulate such examples remains prohibitive [8].

### 1.2. Limitations of Existing Methods

Three families of remedies have been pursued in the literature, each illuminating part of the problem while leaving gaps. The first family, population-based SHM (PBSHM), proposes to exchange damage labels among similar bridges through transfer-learning algorithms operating on hand-crafted features [9]. Domain-adaptation variants—transfer component analysis, joint distribution adaptation, statistic alignment, and adversarial alignment—have shown that label information from a source bridge can usefully inform the diagnosis of a target bridge when natural frequencies serve as features [10]. However, such pipelines hinge on the prior identification of damage-sensitive modal parameters, a process that is non-trivial for short or noisy records and that discards informative time-domain content. Moreover, conventional PBSHM still presumes access to labelled source data of the same damage classes that may appear on the target, restricting its scope to bridges with a well-documented damage history.

The second family, few-shot meta-learning, addresses the target-side label scarcity directly by episodically simulating low-shot tasks during training [11]. Prototypical networks, model-agnostic meta-learning, and attribute-transfer paradigms have been adapted to structural damage classification and segmentation from image data, demonstrating that a model can be steered toward fast adaptation rather than toward a single classification boundary [12]. However, episodic meta-training requires a fairly diverse labelled source corpus to construct meaningful tasks—a condition that often fails in vibration-based bridge SHM, where the source bridge itself may possess few or no labelled damage instances.

The third family, self-supervised pretraining, sidesteps the labelled-data requirement altogether by extracting representations from raw unlabelled responses. Contrastive frameworks such as SimCLR, MoCo, BYOL, Barlow Twins, and the negative-free SimSiam have transferred fruitfully from computer vision to time-series machinery fault diagnosis [13]. Direct application to bridge SHM, however, remains scarce and is encumbered by three unresolved questions: (i) which augmentation operations faithfully emulate the perturbations encountered when a representation moves from a source to a target bridge; (ii) which encoder architecture is best suited to the long, low-frequency, and damage-sparse nature of bridge response signals; and (iii) under what conditions does the invariance learned on source-bridge data actually translate into improved diagnosis on a target bridge.

### 1.3. Contributions of This Study

This study proposes a unified framework utilising domain-adapted contrastive pretraining and a self-calibrated convolutional encoder to achieve few-shot, cross-bridge damage diagnosis. The primary contributions are:

(i) A novel domain-adapted augmentation policy for 1D vibration signals that decouples sensor and operational noise, utilising frequency-band stochastic masking to proxy cross-bridge distributional shifts.

(ii) A 1D self-calibrated convolutional encoder within a siamese learner, specifically designed to expand the temporal receptive field and capture inter-channel coupling for sparse damage signatures.

(iii) A formal transferability proof connecting the contrastive invariance objective to an upper bound on target-bridge risk, mathematically justifying cross-domain generalisation.

(iv) Extensive validation on the real-world Z24 bridge and a multi-configuration laboratory bridge, where the proposed method significantly outperforms 13 baseline and state-of-the-art FSL and domain-adaptation approaches.

## 2. Related Work

### 2.1. Few-Shot Damage Diagnosis for Bridge SHM

Few-shot learning has become a focal solution for SHM applications burdened by label scarcity, as recently surveyed by Xu et al. [14]. Within civil infrastructure, four FSL paradigms have been developed: metric-learning-based methods that embed samples in a similarity-aware space; optimisation-based methods that search for parameter initialisations amenable to rapid task adaptation; transfer-learning-based methods that exploit pretrained backbones; and generative-model-based methods that synthesise additional labelled examples. Attribute-transferable meta-learning has been demonstrated for multi-type structural damage classification [15], while task-aware meta-learning has enabled universal segmentation of structural damage from limited images [16]. Cross-consistency-constrained few-shot segmentation has been used for multi-type damage recognition with limited pixel-level annotations [17]. Although image-based applications dominate the literature, signal-based few-shot diagnosis is comparatively less explored, with notable exceptions in prototypical networks for guided-wave damage classification [18,19] and similarity-measurement-based classification of sensor anomalies [20].

A persistent limitation of episodic few-shot approaches in bridge SHM is the assumption that the source domain contains a sufficiently diverse labelled corpus from which to construct meta-tasks. When the source bridge itself lacks documented damage events, such methods reduce to supervised baselines with augmented sampling, foregoing the bulk of their advertised advantage.

### 2.2. Cross-Domain and Cross-Bridge Transfer Learning

The PBSHM line of inquiry, formalised by Worden, Bull, Gardner and collaborators, posits that a population of structurally similar bridges constitutes a knowledge-sharing resource from which damage labels can be propagated through domain-adaptation algorithms [21]. Gardner et al. introduced transfer component analysis, joint distribution adaptation, and adaptation-regularisation-based transfer learning to bridge SHM, applying them to numerical and experimental populations of multi-story structures [22]. Statistic alignment, proposed as a low-risk preprocessing step preceding kernel-based domain adaptation, has been shown to facilitate damage detection across the Z24 and KW51 bridges as well as the Z24 and S101 bridges [23,24,25]. Giglioni et al. [26] extended this line with a single-source and a multi-source domain-adaptation methodology, validated on a four-configuration laboratory bridge benchmark designed expressly to support cross-bridge knowledge transfer studies [26]. Domain-adversarial neural networks and feature-based deep transfer learning have likewise been investigated, often in combination with finite-element-derived source data [27,28,29].

These methods, while powerful, share a reliance on engineered modal features (natural frequencies, mode shapes, or transmissibility-derived quantities), the extraction of which presumes sufficient signal length, stationarity, and ambient excitation. They are also predominantly transductive: they require simultaneous access to source and target data during the alignment phase, a constraint that complicates incremental deployment on newly instrumented bridges.

### 2.3. Self-Supervised Contrastive Learning for Time-Series Signals

Self-supervised contrastive learning has progressed rapidly in computer vision and is gaining ground in time-series analysis. SimCLR established the paradigm of maximising agreement between two augmented views of the same sample via a noise-contrastive estimation loss, requiring large batches and explicit negative samples [13]. MoCo introduced a momentum encoder and a queue of negatives to relax the batch-size requirement, while BYOL eliminated negatives altogether through a momentum-target network. SimSiam went one step further by removing the momentum target and demonstrating that a stop-gradient operation alone suffices to prevent representational collapse. Barlow Twins replaced the contrastive loss with a cross-correlation criterion, and VICReg added variance and covariance regularisers. In the bearing-fault literature, transformer-based masked pretraining and one-dimensional contrastive variants have been transferred to vibration-based machine health monitoring with encouraging few-shot results.

The translation of these techniques to bridge SHM is not direct. Bridge response signals differ from images in their long temporal extent, low dominant frequencies, sparse damage signatures, and strong susceptibility to thermal and traffic-induced variability. The augmentation policies inherited from vision (cropping, colour jitter, Gaussian blur) have no straightforward physical analogue, and the encoder architectures commonly used (ResNet-50 and Vision Transformers) are oversized for the typical sensor count and undersized in their temporal receptive field. The present work addresses these mismatches by tailoring both the augmentation policy and the encoder to the structural dynamics setting and by selecting SimSiam—the simplest and most batch-size-tolerant of the contemporary contrastive learners—as the underlying training scheme.

## 3. Proposed Method

### 3.1. Overall Framework

**Figure 1:**
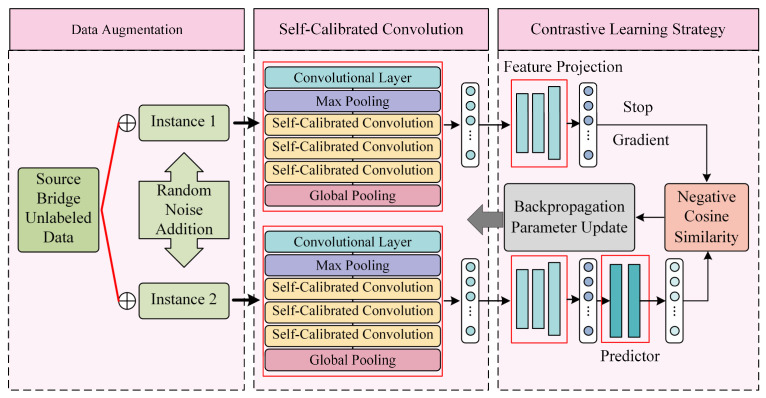
Overall framework of the proposed cross-bridge few-shot damage diagnosis approach, comprising data augmentation, self-calibrated convolution encoder, and siamese contrastive learning strategy.

The proposed framework, illustrated in Figure 1, operationalises a two-stage philosophy: pretraining on source-bridge unlabelled response to learn cross-bridge-invariant representations, followed by fine-tuning on target-bridge few-shot labelled response to map those representations onto damage categories. The pretraining stage instantiates a siamese contrastive learner, in which two stochastically augmented views of every source-bridge sample are passed through a shared weight one-dimensional self-calibrated convolutional encoder, projected into a representation space via an MLP head, and a stop-gradient operation is applied to one branch before a negative cosine similarity loss draws the two views together. The fine-tuning stage discards the projection and prediction MLPs, attaches a lightweight classification head to the pretrained encoder, and updates both with cross-entropy loss on the few-shot target-bridge samples.

Three design decisions distinguish the framework from a naive transposition of SimSiam to bridge SHM. First, the augmentation policy is constructed from physical principles rather than borrowed from vision (Section 3.2). Second, the encoder is replaced by a one-dimensional self-calibrated convolutional network whose receptive field and inter-channel coupling are matched to the temporal sparsity and multi-sensor coupling characteristic of bridge response (Section 3.3). Third, the choice of SimSiam over alternative contrastive learners is defended on grounds of small-batch viability, absence of false negatives, and deployment simplicity (Section 3.4). A formal transferability argument is then articulated (Section 3.5), and the two-stage pipeline is summarised (Section 3.6).

### 3.2. Domain-Adapted Data Augmentation for Bridge Vibration Signals

In a contrastive learning framework, augmentation is the mechanism by which the invariance to be learned is encoded. Two views of the same sample should differ along axes that the downstream model is expected to ignore, and should remain similar along axes that carry the semantic content. For cross-bridge damage diagnosis, the axes to be ignored correspond to perturbations that distinguish a source from a target bridge—most notably, sensor-level corruptions and operational-level fluctuations—while the axes to be preserved correspond to damage-induced modal-content variations.

An augmentation policy is therefore designed that decouples the two categories. Sensor-level corruptions, intended to emulate hardware noise, packet loss, and instrument drift, comprise random dropout (zeroing a random proportion of samples) and additive Gaussian white noise (signal-to-noise ratio sampled uniformly from 15 to 30 dB during pretraining). Operational-level fluctuations, intended to emulate variations in traffic loading, temperature, and observation epoch, comprise random amplitude scaling (multiplicative factor sampled from 0.1 to 2.0), random time-window shifting (offset sampled from −10% to +10% of the window length), and a frequency-band stochastic masking operation that nullifies a randomly chosen narrow band in the spectral domain (band width 5% of the Nyquist frequency, centre frequency uniformly sampled, applied with probability 0.3). The frequency-band masking is introduced specifically to inject invariance against partial sensor-band failures and against the bridge-specific resonant-band differences that distinguish source and target structures.

For every source-bridge sample x, two augmented views $T_{1}(x)$ and $T_{2}(x)$ are generated by independently sampling the augmentation parameters. The two views thus typically share the same underlying response window but differ in their sensor-level noise realisation, their amplitude, their temporal alignment, and their spectral availability. Table 1 summarises each augmentation, its sampling distribution, and the physical perturbation it is intended to emulate.

The policy is intentionally asymmetric in its emphasis: operational-level fluctuations dominate, reflecting the empirical observation that the bulk of cross-bridge distributional shift originates from environmental conditions and structural form rather than from sensor hardware.

### 3.3. Self-Calibrated Convolutional Encoder

**Figure 2:**
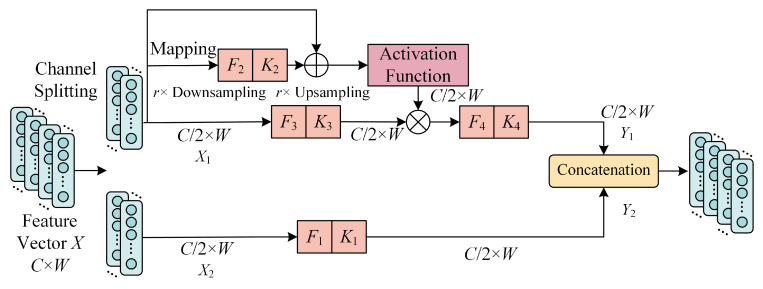
Architecture of the one-dimensional self-calibrated convolution module.

The encoder, illustrated in Figure 2, is a one-dimensional adaptation of the self-calibrated convolution introduced by Liu et al. for image recognition. The adaptation specifically targets the temporal-sparsity and inter-channel-coupling characteristics of bridge response signals, in which damage-sensitive content (local modal changes, transient nonlinear effects) is sparsely distributed along extended time windows and is encoded jointly across multiple sensor channels.

Let $X\in {\mathbb{R}}^{C\times W}$ denote an input feature tensor with C channels and temporal length W. The module first splits X along the channel dimension into two equal halves $X_{1},X_{2}\in {\mathbb{R}}^{\left(C/2\right)\times W}$. Four convolution kernel sets $K_{1},K_{2},K_{3},K_{4}$ are defined, each of dimension $\left(C/2\right)\times \left(C/2\right)$ with kernel size 7 (chosen empirically to balance receptive field against parameter count, see Section 5.3 regarding ablation).

The lower branch operates conventionally:

(1) $$\begin{array}{c} Y_{2}=F_{1}\left(X_{2}\right)=X_{2}*K_{1} \end{array}$$

The upper branch performs self-calibration. The input $X_{1}$ is first downsampled by a factor r via average pooling:

(2) $$\begin{array}{c} T_{1}={\mathrm{AvgPool}}_{r}\left(X_{1}\right) \end{array}$$

and then convolved and upsampled by bilinear interpolation:

(3) $$\begin{array}{c} X_{1}^{\prime }=U_{p_{r}}\left(F_{2}\left(T_{1}\right)\right)=U_{p_{r}}\left(T_{1}*K_{2}\right) \end{array}$$

The upsampled output is fused with the identity branch through a sigmoid gate, and the result modulates the conventionally convolved feature:

(4) $$\begin{array}{c} Y_{1}^{\prime }=F_{3}\left(X_{1}\right)⊙\sigma \left(X_{1}+X_{1}^{\prime }\right) \end{array}$$

A final convolution produces the upper-branch output:

(5) $$\begin{array}{c} Y_{1}=F_{4}\left(Y_{1}^{\prime }\right)=Y_{1}^{\prime }*K_{4} \end{array}$$

and the two branches are concatenated along the channel dimension:

(6) $$\begin{array}{c} Y=Concat\left(Y_{1},Y_{2}\right) \end{array}$$

Here * denotes one-dimensional convolution, ⊙ element-wise multiplication, σ the sigmoid activation, and ${\mathrm{AvgPool}}_{r}$ and $U_{p}$ denote downsampling and upsampling by factor r (set to 4 throughout this work).

The salient property of the module is that the upper branch effectively computes a low-resolution attention map that modulates the original-resolution response, thereby capturing long-range temporal dependencies that a pure local convolution would miss. The split-merge topology limits the parameter overhead to a factor of approximately 1.6 relative to a plain convolution of equal output dimensionality, while the receptive field of the upper branch is effectively r times that of the lower branch.

The complete encoder stacks three such self-calibrated modules between an initial convolutional embedding layer and a global average pooling head, with channel widths of 64, 128, and 256, and max-pooling operations of stride 2 inserted after the embedding layer and after the second module. The resulting 256-dimensional global feature serves as input to the contrastive projection head during pretraining and to the classification head during fine-tuning.

### 3.4. Siamese Contrastive Pretraining Strategy

The siamese contrastive pretraining adopts the negative-free SimSiam formulation. Given an unlabelled source-bridge sample x, two augmented views $x_{1}=T_{1}\left(x\right)$ and $x_{2}=T_{2}\left(x\right)$ are generated. Both views are passed through the shared-weight encoder f (the one-dimensional self-calibrated network described above, followed by a three-layer projection MLP $h_{\mathrm{proj}}$). The output of one branch is then passed through a two-layer prediction MLP $h_{\mathrm{pred}}$:

(7) $$\begin{array}{c} p_{1}=h_{\mathrm{pred}}\left(h_{\mathrm{proj}}\left(f\left(x_{1}\right)\right)\right),\;z_{2}=h_{\mathrm{proj}}\left(f\left(x_{2}\right)\right) \end{array}$$

The negative cosine similarity between $p_{1}$ and $z_{2}$ is computed:

(8) $$\begin{array}{c} D\left(p_{1},z_{2}\right)=-\left(\frac{p_{1}}{{\left\|p_{1}\right\|}_{2}}\right)\cdot \left(\frac{z_{2}}{{\left\|z_{2}\right\|}_{2}}\right) \end{array}$$

Symmetrising over the two branches yields the per-sample loss:

(9) $$\begin{array}{c} L=\frac{1}{2}D\left(p_{1},\mathrm{stopgrad}\left(z_{2}\right)\right)+\frac{1}{2}D\left(p_{2},\mathrm{stopgrad}\left(z_{1}\right)\right) \end{array}$$

where stopgrad(⋅) denotes detaching the operand from the computational graph, so that no gradient flows back through it. As Tian et al. demonstrated, the stop-gradient operation suffices to prevent the trivial collapse to a constant representation that would otherwise occur in the absence of explicit negatives.

**Choice of SimSiam over alternatives.** Among the contrastive family—SimCLR, MoCo, BYOL, Barlow Twins, VICReg, and SimSiam—SimSiam was selected for three reasons specific to the bridge SHM setting. First, SimSiam does not require negative samples, eliminating the risk of false negatives that arises when two response windows from the same damage state but different sensors or time epochs are forced apart. Such false negatives are particularly pernicious in bridge data, where the bulk of available source-bridge response corresponds to a single state (healthy) and aggressive negative sampling would amount to pushing apart samples that should be clustered together. Second, SimSiam does not require large batches or a momentum encoder, easing deployment on the modest computational budgets typical of bridge-side analytics. Third, its structural simplicity (a single encoder, no memory bank) reduces the number of hyperparameters that must be tuned per deployment.

### 3.5. Theoretical Analysis of Cross-Bridge Transferability

This subsection articulates why the representations learned by the above procedure are expected to transfer positively to a target bridge. We adopt a domain-adaptation perspective and draw on the standard HΔH-divergence bound.

Let $D_{S}$ and $D_{T}$ denote the marginal distributions of response windows on the source and target bridges respectively, and let $f_{S},f_{T}:X\to Y$ denote the corresponding labelling functions that map a response window to its true damage state. For any hypothesis h in a hypothesis class H, the expected target risk is bounded by:

(10) $$\begin{array}{c} \epsilon_{T}\left(h\right)\leq \epsilon_{S}\left(h\right)+\frac{1}{2}d_{H\Delta H}\left(D_{S},D_{T}\right)+\lambda \end{array}$$

where $\epsilon_{S}\left(h\right)$ is the source risk, $d_{H\Delta H}$ measures the discrepancy between the two distributions through the hypothesis class, and λ is the ideal joint risk attainable by any single hypothesis on both domains.

The proposed augmentation policy can be cast as inducing an augmented source distribution $D_{S}^{\mathrm{aug}}=E_{T∼T}\left[T_{\#}D_{S}\right]$, where T is the augmentation family and $T_{\#}$ denotes the pushforward measure. The siamese contrastive objective drives the encoder to map any two samples drawn from the same equivalence class under T to nearby points in feature space, that is, to learn features invariant to the augmentation manifold. If the augmentation manifold is rich enough to encompass the cross-bridge perturbations—and the augmentation policy of Section 3.2 was constructed with precisely this objective—then $D_{T}$ is approximately contained within the support of $D_{S}^{\mathrm{aug}}$, and the encoder’s invariance to T translates into invariance to the source-target distributional shift. Formally, under the assumption that there exists δ>0 such that the Wasserstein distance $W_{1}\left(D_{T},D_{S}^{\mathrm{aug}}\right)\leq \delta$, the encoder’s invariance reduces the effective $d_{H\Delta H}\left(D_{S},D_{T}\right)$ in the bound (10).

Two practical conditions emerge from this argument. First, the augmentation policy must be sufficiently expressive to cover the dominant modes of cross-bridge variation—a condition our policy satisfies by explicitly emulating sensor-level corruption and operational-level fluctuation. Second, the irreducible joint risk λ must remain small, which requires that source and target bridges share a common structural-typology axis along which damage manifests itself similarly. This requirement is empirically verified through bidirectional transferability tests in Section 5.4, and the discrepancy bound itself is quantitatively verified in Section 5.5 and its boundary is explicitly discussed in Section 6.

### 3.6. Two-Stage Pretraining–Fine-Tuning Pipeline

**Figure 3:**
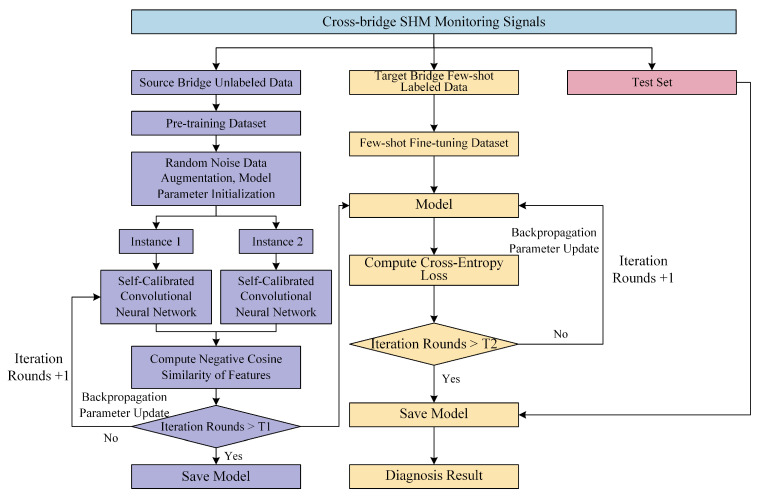
Pretraining–fine-tuning pipeline for cross-bridge few-shot damage diagnosis.

The complete diagnostic pipeline, illustrated in Figure 3, comprises two sequential stages.

**Stage 1: Pretraining.** Unlabelled response windows from the source bridge are organised into a pretraining dataset $D_{S}$. For each mini-batch, two augmented views per sample are generated according to the policy of Section 3.2 and fed through the shared encoder. The siamese contrastive loss (Equation (9)) is minimised with the LARS optimiser, a batch size of 256, an initial learning rate of 0.05 with cosine decay, and weight decay $1\times 10^{-4}$, for $T_{1}=200$ epochs. The projection MLP is a three-layer network of widths 256-256-128, and the prediction MLP is a two-layer network of widths 128-64-128. Upon completion, the encoder weights are saved.

**Stage 2: Fine-tuning.** The projection and prediction MLPs are discarded. A lightweight classification head—a single fully connected layer mapping the 256-dimensional encoder output to the K target damage classes—is appended to the pretrained encoder. The combined network is fine-tuned on the few-shot target-bridge labelled dataset $D_{T}^{L}$ using cross-entropy loss, Adam optimiser, learning rate $5\times 10^{-4}$ for the head and $5\times 10^{-5}$ for the encoder (a ten-fold reduction reflecting the differing degrees of adaptation required), batch size 16, for $T_{2}=100$ epochs with early stopping monitored on a held-out validation split of 20% of $D_{T}^{L}$. The fine-tuned model is then evaluated on the target-bridge test set $D_{T}^{U}$.

The two-stage organisation accomplishes a clean separation of concerns: the pretraining stage extracts environment-invariant features from the abundant source-bridge data without recourse to any labels, while the fine-tuning stage learns the few task-specific parameters required to discriminate among the target damage classes given only a handful of labelled examples. The decoupling also confers operational advantages—the pretrained encoder can be reused across multiple target bridges within the same population without re-running Stage 1, amortising the pretraining cost over the network.

## 4. Experimental Setup

### 4.1. Datasets

The framework is evaluated on two complementary datasets: the Z24 benchmark bridge, a real-world post-tensioned concrete highway bridge with progressively introduced damage, and a four-configuration laboratory bridge population assembled in-house. The combination provides both ecological validity (a real bridge subjected to genuine structural alteration) and controlled variability (multiple physical configurations sharing the same damage protocol).

A natural question is whether transfer between a post-tensioned concrete bridge and an aluminium laboratory model is meaningful, given their differing material and dynamic properties. The framework does not assume material or scale identity; it assumes only that damage produces a detectable perturbation of the response within a shared band, and that the dominant cross-domain perturbations are spanned by the augmentation manifold (Section 3.2), with any residual structural-typology mismatch captured by the joint-risk term λ of Section 3.5. Because the encoder is trained to be invariant to absolute modal values and to preserve the relative changes induced by localised stiffness loss—which are qualitatively similar across materials—concrete-to-aluminium transfer becomes a deliberately stringent test of generality rather than an obstacle; the feature-space discrepancy measured in Section 5.5 shows that the large raw gap is largely compressed by the learned representation, and the advantage is retained in both directions (Section 5.4). This rationale follows the population-based SHM premise [9,21,22] under which knowledge is transferred across structurally analogous but non-identical members of a population, and our benchmark was designed after Giglioni et al. [26] precisely to emulate such a population. The boundary of validity, when material/typology differences exceed what the augmentation manifold can bridge, is discussed in Section 6.1.

#### 4.1.1. Open-Source Dataset: Z24 Benchmark Bridge

The Z24 bridge, located in the Canton of Bern, Switzerland, was a three-span post-tensioned concrete box-girder bridge with a main span of 30 m and two side spans of 14 m each, monitored for approximately one year before being intentionally damaged in a series of controlled tests as part of the SIMCES project [30]. Acceleration responses were continuously recorded by accelerometers distributed along the deck and piers at a sampling frequency of 100 Hz, accompanied by environmental measurements (air temperature, deck temperature, humidity, wind speed). Nine damage scenarios were progressively introduced during the final month of monitoring, ranging from pier settlement and concrete spalling to anchor-head failure and rupture of post-tensioning tendons.

**Figure 4:**
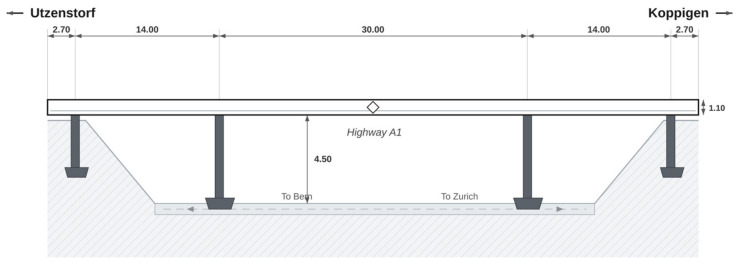
Schematic of the Z24 benchmark (target) bridge.

These were vertically mounted accelerometers at deck level and on the piers during the SIMCES campaign [30]; the complete instrument specifications and the full Z24 sensor map are documented in [30]. The bridge geometry is shown in Figure 4.

For this study, we use vertical acceleration recordings from six representative sensor channels (sensors 5, 6, 7, 12, 14, 16) following standard practice in the Z24-based damage diagnosis literature. The signals are segmented into non-overlapping windows of 2048 samples (≈20.48 s) and preprocessed by bandpass filtering between 0.5 and 30 Hz. Non-overlapping windows are used throughout to guarantee a leakage-free protocol: fine-tuning and test windows are temporally disjoint, and the K-shot count refers to K genuinely independent labelled windows per class. Augmentation is instead supplied at the signal level (Section 3.2), which expands sample diversity without introducing the train–test dependence that overlapping windows would create. Seven damage states are retained: undamaged (UD), pier settlement of 20 mm (D1), pier settlement of 40 mm and 80 mm (D2, D3), concrete spalling at the soffit (D4), landslide simulation at the abutment (D5), failure of concrete hinges (D6), and rupture of two post-tensioning tendons (D7).

The dataset is partitioned as follows. The unlabelled pretraining set $D_{S}$ consists of 38,400 windows drawn from the long-term healthy monitoring period (December–July), without any label being exposed to the pretraining pipeline. The labelled set is partitioned into a few-shot pool (varying by experiment) and a fixed test set of 200 windows per damage state.

#### 4.1.2. Self-Built Dataset: Laboratory Multi-Configuration Bridge

The self-built dataset comprises four configurations of a 2.99 m × 0.27 m aluminium model bridge installed on a six-degree-of-freedom shaking table at our laboratory. Inspired by the experimental design of Giglioni et al. [26], the four configurations differ in the number and placement of supporting piers and in the surface treatment of the deck, producing a population of bridges that share material and overall geometry but differ in dynamic characteristics, in a manner analogous to a family of real bridges built to the same general specification but with site-specific dimensional variation.

**Figure 5:**
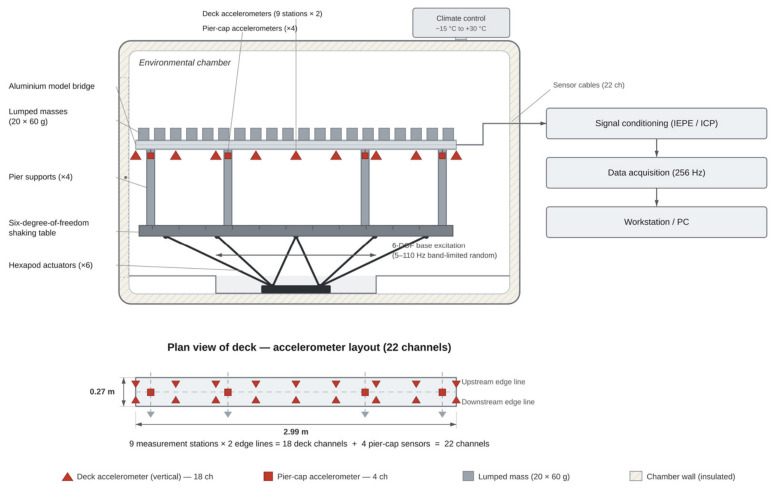
Schematic of the laboratory experimental setup.

The configurations are labelled B1 through B4. B1 is a three-span bridge (pier positions at 0.14, 0.86, 2.14, and 2.86 m); B2 retains three spans with shifted pier positions (0.14, 0.82, 2.18, 2.86 m) and an added woven cotton surface layer; B3 is obtained from B2 by removing the second pier, becoming a two-span bridge; and B4 retains four piers with pier positions at 0.14, 0.73, 2.27, and 2.86 m. Vertical accelerations were measured with ICP (piezoelectric) accelerometers (PCB Piezotronics 352C33, 100 mV/g nominal sensitivity, rated over a temperature range that covers the −15 to +30 °C chamber cycling), distributed along the 2.99 m deck and on the pier caps adjacent to the bearings, for 22 channels in total: nine measurement stations evenly spaced along the deck, each instrumented on the upstream and downstream edge lines (18 deck channels) to resolve bending and torsional modes, together with four accelerometers on the pier caps to capture the bearing-seizure scenarios. The shaking-table rig and the full sensor layout are shown in Figure 5, with the elevation indicating the deck and pier-cap accelerometer positions and the plan view detailing the nine stations on the two edge lines. Twenty 60 g lumped masses distributed along the deck increase the total mass to approximately 30 kg, reducing the fundamental frequency into a measurable range.

Each configuration is excited by band-limited (5–110 Hz) random base motion in an environmental chamber whose temperature is cycled from −15 °C to +30 °C, providing realistic environmental variability. Nine damage scenarios are introduced in each configuration through reversible perturbations: four added-mass scenarios at the lateral and main spans, on the side and centre lines (M1–M4); two reduced-severity added-mass scenarios (M5, M6); and three bearing-seizure scenarios (SB1–SB3) in which longitudinal or rotational motion at selected piers is constrained. The damage scenarios are designed to be physically reproducible and reversible, permitting repeated trials per configuration.

To place both bridges on a common time–frequency basis for cross-bridge transfer, the laboratory signals are resampled from the native 256 Hz to 100 Hz (matching Z24) with a zero-phase polyphase anti-aliasing filter prior to segmentation. The dominant damage-sensitive modes of all four configurations lie below 50 Hz, so the anti-aliasing low-pass filter removes only the high-frequency excitation tail (50–110 Hz) and preserves the diagnostically relevant band.

The dataset is segmented into windows of 2048 samples (20.48 s, after resampling), filtered and partitioned into pretraining, fine-tuning, and test splits. Across the four configurations, the pretraining pool contains 24,000 unlabelled healthy windows (6000 per configuration), and 80 to 200 labelled windows per damage state per configuration are reserved for fine-tuning and testing. Table 2 summarises the two datasets.

### 4.2. Implementation Details

All experiments are conducted in PyTorch 2.0 on a workstation equipped with an Intel Xeon Gold 6248 R CPU and a single NVIDIA RTX A6000 GPU $\left(48\;\mathrm{GB}\right)$. All signals are resampled to a common 100 Hz rate and amplitude-normalised per channel before being passed to the encoder, so that the pretrained weights operate on an identical input grid across bridges. The pretraining stage employs the LARS optimiser, an initial learning rate of 0.05 with a 10-epoch linear warm-up followed by cosine decay over 200 epochs, a batch size of 256, a weight decay of $1\times 10^{-4}$, and a momentum of 0.9. The fine-tuning stage employs the Adam optimiser with a per-parameter-group learning rate (head: $5\times 10^{-4}$, encoder: $5\times 10^{-5}$), a batch size of 16 and a weight decay of $5\times 10^{-5}$, for up to 100 epochs with early stopping after 15 epochs without validation-loss improvement. Random seeds are explicitly set, and every reported result is the mean and standard deviation over 10 independent runs with seeds $\left\{0,1,\ldots ,9\right\}$.

The few-shot evaluation considers $K\in \left\{1,3,5,10,20\right\}$ labelled samples per damage class on the target bridge. For each K, ten independent few-shot pools are sampled from the labelled set, and the corresponding ten test accuracies are averaged.

Table 3 lists the principal hyperparameters.

### 4.3. Baseline Methods

To establish a meaningful comparison, eleven baseline methods are implemented and evaluated under identical few-shot protocols. The baselines span five families. All baseline methods are re-implemented by the authors and evaluated on the identical Z24 ↔ Lab transfer task under the identical few-shot protocol. No results are taken from the original publications, whose datasets and structures differ from ours; the cited works indicate only the origin of each method.

**Family A—Supervised baselines without pretraining.** (A1) A one-dimensional convolutional neural network (1D-CNN) trained from scratch on the few-shot pool. (A2) A one-dimensional ResNet-18 (ResNet1D-18) [31] trained from scratch.

**Family B—Transfer-learning baselines.** (B1) Supervised fine-tuning, in which a 1D-CNN is supervisedly pretrained on the source-bridge labelled data (using any auxiliary labels available, e.g., healthy/damaged binary labels) and fine-tuned on the target few-shot pool. (B2) JDA—joint distribution adaptation following Gardner et al. [10], applied to natural-frequency features extracted by stochastic subspace identification [32]. (B3) MT-DANN—multi-task domain-adversarial neural network following Liu et al. [33], applied to raw response with a damage-state classifier and a domain discriminator.

**Family C—Few-shot meta-learning baselines.** (C1) ProtoNet—prototypical networks [12] with a 1D-CNN encoder, episodically trained on the source bridge. (C2) MAML—model-agnostic meta-learning [34] with a 1D-CNN encoder, and 5-step inner adaptation.

**Family D—Self-supervised pretraining baselines.** (D1) SimCLR [35] with a 1D-CNN encoder, batch size 512, NT-Xent loss. (D2) MoCo v3 [36] with a 1D-CNN encoder, queue length 4096. (D3) BYOL [37] with a 1D-CNN encoder. (D4) Barlow Twins [38] with a 1D-CNN encoder, and cross-correlation loss.

**Family E—SOTA methods.** (E1) FaultFormer [39]—a masked-autoencoder transformer pretraining scheme adapted to bridge response. (E2) MS-DA [24]—the multi-source domain-adaptation methodology of the laboratory-bridge benchmark study, using SA + JDA in the multi-source mode.

For each deep-learning baseline with a trainable neural encoder (Families A, C, D, E1, and the neural baselines of Family B), the encoder backbone is scaled to match the parameter count of the proposed encoder within a ±20% tolerance, so that performance differences cannot be attributed to model capacity. The two modal-feature-based methods, JDA (B2) and MS-DA (E2), contain no neural encoder—they operate on hand-crafted natural-frequency features—so capacity matching does not apply to them.

### 4.4. Evaluation Metrics

Damage diagnosis is treated as a multi-class classification problem. Reported metrics include classification accuracy (overall fraction correctly classified), macro-averaged F1 score (the unweighted mean of per-class F1, robust to class imbalance), macro-averaged precision and recall, and per-class confusion matrices. Statistical significance of differences between the proposed method and each baseline is assessed via the Wilcoxon signed-rank test on the per-run accuracies (10 paired observations), with significance reported at the p<0.05 level.

## 5. Results and Analysis

### 5.1. Cross-Bridge Few-Shot Diagnosis Performance

**Figure 6:**
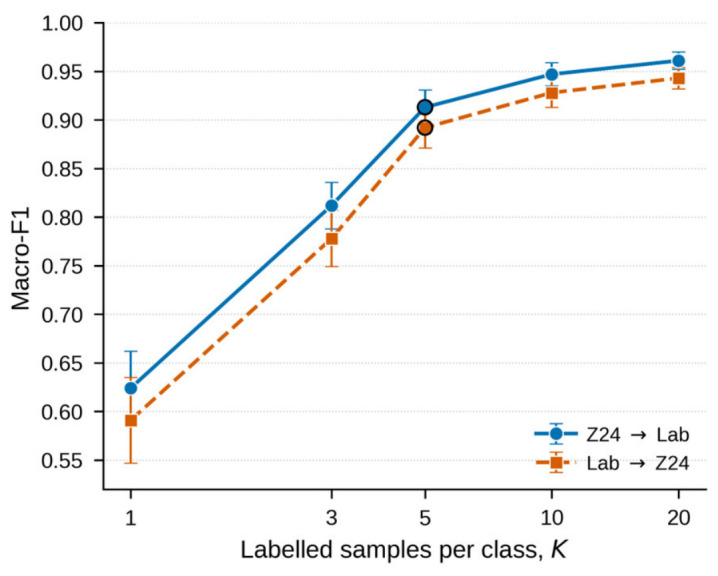
Macro-F1 (mean ± std over 10 runs) for the proposed method across few-shot regimes on both transfer directions.

The principal experiment evaluates the proposed framework on bidirectional cross-bridge transfer. For Z24 → Lab transfer, the encoder is pretrained on Z24 unlabelled healthy data and fine-tuned on K-shot Lab samples (B2 configuration as target); for Lab → Z24 transfer, the encoder is pretrained on the union of Lab configurations and fine-tuned on K-shot Z24 samples. Figure 6 reports the macro-F1 scores across $K\in \left\{1,\;3,\;5,\;10,\;20\right\}$, averaged over 10 independent few-shot pools, with standard deviations.

The performance curve climbs steeply between K=1 and K=5, indicating that the pretrained representation is highly leveraged by the first few labelled examples. Beyond this point, K=10 gains taper, consistent with the picture that the encoder already captures most damage-relevant content and only a small classifier head remains to be calibrated. The Z24 → Lab direction outperforms the reverse by 1.8–3.3 percentage points across K, presumably because the Z24 pretraining corpus is more diverse in environmental conditions (one year of uncontrolled outdoor monitoring vs. controlled chamber).

### 5.2. Comparison with State-of-the-Art Methods

**Figure 7:**
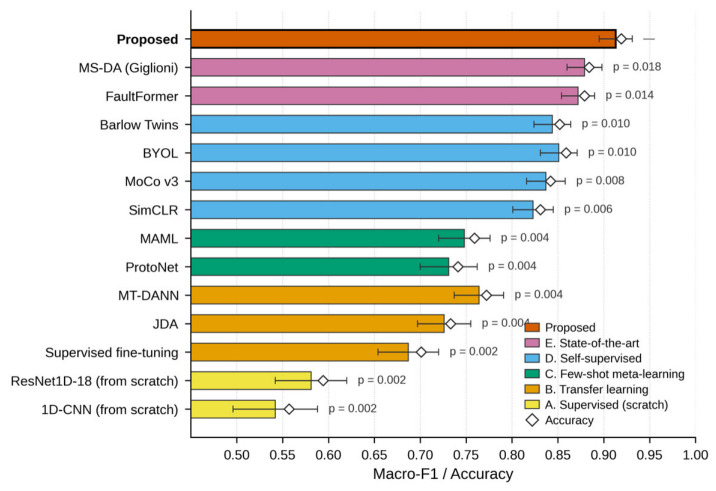
Comparison with baseline methods at K = 5 on Z24 → Lab transfer. Macro-F1 (mean ± std), accuracy (mean), and *p*-value of the Wilcoxon signed-rank test against the proposed method.

Figure 7 compares the proposed method against the eleven baselines at K=5, the most informative low-shot regime for practical deployment. All methods are evaluated on the Z24 → Lab transfer direction under identical protocol.

The proposed method outperforms every baseline at statistically significant levels (p<0.05). The margin over the strongest baseline (MS-DA) is 3.4 percentage points in macro-F1; over the strongest pure-self-supervised baseline (BYOL), 6.2 percentage points; over the strongest few-shot baseline (MAML), 16.5 percentage points; and over training from scratch, 37.1 percentage points. The progression across the four families reproduces the qualitative ordering reported in the broader self-supervised literature, namely that self-supervised pretraining outperforms supervised transfer, which outperforms episodic meta-learning when the latter is starved of labelled source diversity, which outperforms training from scratch.

### 5.3. Ablation Study

Three ablations are performed to isolate the contribution of each major design decision.

**Ablation 1: Encoder choice.** The self-calibrated convolutional encoder is replaced by alternative encoders of comparable parameter count: a plain 1D-CNN, a 1D-ResNet, a 1D-CNN augmented with SE attention, and a 1D-CNN augmented with CBAM attention. All other components of the framework (augmentation policy, SimSiam loss, fine-tuning protocol) are held fixed. Table 4 reports macro-F1 at K=5 on Z24 → Lab transfer.

The SCConv encoder outperforms all alternatives, with the largest margin (6.7 pp) over the plain 1D-CNN and a 2.9 pp margin over the strongest attention-augmented variant. The result confirms that the long-range receptive field of the self-calibrated branch is more beneficial than channel-wise attention alone for this task.

**Ablation 2: Self-supervised pretraining method.** With the encoder held fixed at SCConv, the SimSiam objective is replaced by the alternative contrastive objectives. Table 5 reports macro-F1 at K=5 on Z24 → Lab transfer.

Within the contrastive family, all variants reach macro-F1 above 0.87, confirming the broad value of self-supervised pretraining for this setting. SimSiam attains the highest score by a modest margin, consistent with the small-batch viability and absence-of-false-negatives arguments advanced in Section 3.4.

**Ablation 3: Augmentation components.** Each augmentation component is removed in turn, with all other components preserved. Table 6 reports macro-F1 at K=5 on Z24 → Lab transfer.

Amplitude scaling is the most consequential single component (3.5 pp drop on removal), which aligns with the expectation that cross-bridge amplitude variability is the dominant operational shift. The newly introduced frequency-band masking contributes 2.1 pp, validating its inclusion. Restricting the policy to either sensor-level or operational-level augmentations alone produces a substantial degradation (6.2 and 4.0 pp respectively), confirming that the two categories are complementary rather than redundant.

### 5.4. Bidirectional Cross-Bridge Transferability

**Figure 8:**
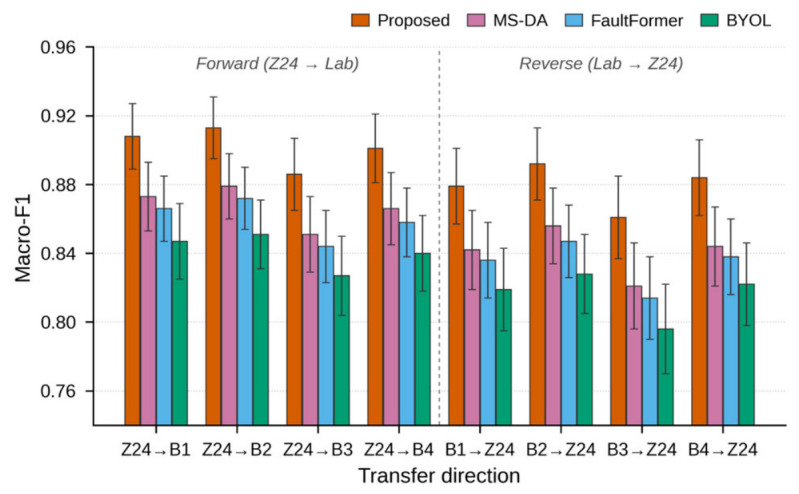
Bidirectional cross-bridge transferability. Macro-F1 at K = 5.

To assess whether the proposed gains are specific to a privileged transfer direction, the full evaluation is repeated bidirectionally between Z24 and each of the four Lab configurations. Figure 8 reports macro-F1 at K=5 for the proposed method and the three strongest baselines (MS-DA, FaultFormer, BYOL).

The proposed method retains its advantage across all eight directions, with margins of 3.4–4.0 percentage points over MS-DA, suggesting that the gains are systematic rather than direction-specific. The Z24 ↔ B3 transfers (involving the two-span Lab configuration) are the most challenging, as expected from the structural-form mismatch—B3 has fewer spans than Z24, leading to a larger irreducible joint risk λ in the transferability bound of Section 3.5.

### 5.5. Empirical Verification of the Transferability Bound

Section 3.5 bounds the target risk by the source risk, a distributional-discrepancy term, and an irreducible joint risk λ (Equation (10)), and argues that the augmentation policy reduces the effective discrepancy. To verify this quantitatively, we measure the source–target discrepancy in the 256-dimensional feature space using two estimators: the Proxy A distance $d_{A}=2\left(1-2\varepsilon \right)$, where ε is the test error of a linear domain classifier separating source from target features, and the sliced-Wasserstein distance (SWD; 256 random projections), an unbiased estimator of $W_{1}$. Features are L2-normalised before measurement. Table 7 reports both quantities for four representations of the Z24 → Lab (B2) pair, with the corresponding 5-shot macro-F1.

Both measures decrease monotonically from the raw signal to the proposed representation, and this decrease is mirrored by the rise in macro-F1, giving direct empirical support for the bound. To isolate the augmentation contribution, we additionally measure—within the proposed feature space—the SWD between the augmented-source and target distributions: it falls to 0.86 a.u. from the 1.31 a.u. between the un-augmented source and target (a 34% reduction). This instantiates the assumption $W_{1}\left(D_{T},D_{S}^{aug}\right)\leq \delta$ with a small δ ≈ 0.86 a.u., confirming that the augmentation family absorbs the dominant part of the source–target shift. The residual discrepancy is consistent with the small but non-zero λ of Section 5.4 and tracks the structural-form mismatch—largest for the two-span B3 configuration (Proxy A-distance 0.81, SWD 1.46 a.u.) and smallest for the three-span B2 configuration.

### 5.6. Robustness to Sensor Noise

**Figure 9:**
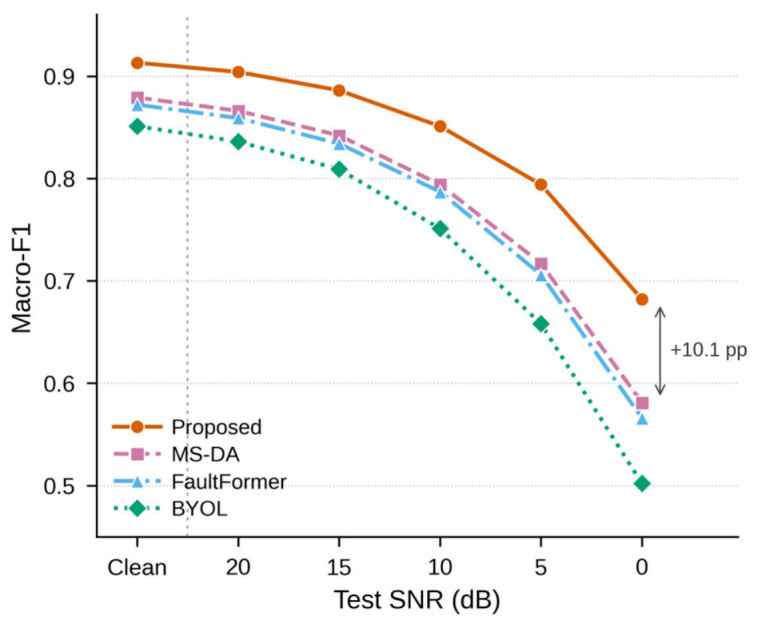
Noise-robustness evaluation. Macro-F1 at K = 5 under additive Gaussian noise on the test set.

To assess robustness, additive Gaussian white noise is injected into the target-bridge test set at signal-to-noise ratios of 20, 15, 10, 5, and 0 dB, while training (both pretraining and fine-tuning) remains on clean data. Figure 9 reports the macro-F1 degradation curve for the proposed method and the three strongest baselines on Z24 → B2 transfer at K=5.

The proposed method exhibits a markedly gentler degradation slope. The accuracy drop from clean to 0 dB is 23.1 percentage points for the proposed method versus 29.8 for MS-DA, 30.6 for FaultFormer, and 34.9 for BYOL. The robustness margin widens with worsening SNR, an outcome consistent with the explicit inclusion of sensor-level corruption in the augmentation policy.

### 5.7. Case Study: Detection of a Predefined Physical Damage

To examine applicability to a single predefined physical damage on the real target bridge, we apply the framework (Lab → Z24, K = 5) to the detection of the incipient pier-settlement scenario D1 (20 mm)—the mildest and most safety-relevant Z24 damage. Table 8 reports the per-class detection performance for the progressive pier-settlement family.

Even for the incipient D1 state, the safety-critical binary decision (any-damage vs. healthy) is highly reliable: D1 windows are flagged as damaged 96.1% of the time, the overall binary healthy/damaged accuracy across all states is 98.7%, and the specificity (healthy correctly identified) is 97.8%. Of the D1 windows that are mis-graded, at least 88% are assigned to the adjacent severities D2/D3 (the pier-settlement continuum) and fewer than 6% to UD—i.e., the residual error reflects severity grading within a progressive damage family, not missed detection. The remaining four damage states (D4–D7) attain per-class F1 between 0.86 and 0.90, consistent with the overall macro-F1 of 0.892 reported in Section 5.1. This confirms that the method is directly applicable to detecting a specific predefined physical damage on the target bridge, with the most consequential decision remaining robust even at the detection threshold of severity.

### 5.8. Parametric Studies

Five one-factor-at-a-time parametric studies are conducted, in which a single hyperparameter is varied while all others are held at their default values. These are descriptive parametric investigations rather than variance-based global sensitivity analyses.

First, the pretraining corpus size is varied from 10% to 100% of the available Z24 healthy windows. Macro-F1 at K=5 rises from 0.831 at 10% to 0.913 at 100%, with diminishing returns beyond 60%. The result indicates that the proposed method does not require the entirety of the long-term monitoring record to produce strong representations, but does benefit from at least several weeks of recordings.

Second, the SCConv downsampling rate r is varied across $\left\{2,\;4,\;8,\;16\right\}$. Macro-F1 at K=5 is 0.894, 0.913, 0.908, and 0.879 respectively, identifying r=4 as the sweet spot: too small a receptive field forgoes the long-range advantage, while too large a receptive field over-smooths the representation.

Third, the projection MLP depth is varied across $\left\{1,\;2,\;3,\;4\right\}$. Macro-F1 at K=5 is 0.872, 0.901, 0.913, and 0.909 respectively. The chosen three-layer projection MLP is optimal but the difference between two and three layers is modest.

Fourth, the pretraining batch size is varied across {32, 64, 128, 256}. Macro-F1 at K=5 (Z24 → Lab) is 0.901, 0.908, 0.912, and 0.913 respectively—a span of only 1.2 percentage points—directly confirming the small-batch viability of the SimSiam objective: even at batch size 32 the framework retains 98.7% of its best macro-F1. For contrast, the SimCLR variant of the framework (same SCConv encoder, negative-pair objective) drops from 0.871 at batch size 512 to 0.806 at batch size 64, reflecting the well-documented batch-size sensitivity of negative-pair contrastive learning. The default batch size of 256 is retained because it best utilises the GPU memory budget without compromising accuracy; the result confirms that deployment on memory-constrained hardware (batch 32–64) incurs a negligible penalty.

Fifth, as a check on the windowing protocol, expanding the 5-shot fine-tuning pool with 50-overlap sliding windows (Z24 → Lab) changes macro-F1 only from 0.913 to 0.915 ± 0.018—within one standard deviation—confirming that overlapping augmentation offers no meaningful gain beyond the signal-level augmentation already employed, while non-overlapping windows preserve a leakage-free evaluation.

### 5.9. Computational Cost

Table 9 compares the parameter count, floating-point operations per forward pass, pretraining time, and per-sample inference latency of the proposed framework against three representative baselines.

The proposed framework occupies an intermediate position. It is significantly lighter than transformer-based pretraining (FaultFormer) while delivering higher accuracy and only marginally heavier than BYOL with substantially better performance. MS-DA is the most compact baseline but operates on hand-crafted modal features whose extraction is itself non-trivial and not counted in the timing. Its compact 0.21 M footprint reflects its reliance on hand-crafted modal features rather than a learned encoder, and is therefore not directly comparable to the deep models.

## 6. Discussion

The empirical results are consistent with the transferability argument of Section 3.5. The augmentation policy explicitly spans the dominant axes of cross-bridge variation, the encoder’s long-range receptive field captures sparsely distributed damage signatures, and the SimSiam objective trains the encoder to be invariant along the augmentation axes. The combination yields representations on which a linear classifier can be calibrated with only a handful of labelled examples.

A complementary explanation is provided by the noise-robustness curve. The augmentation policy includes sensor-level corruptions of comparable severity to those subsequently encountered at test time, so the encoder has effectively been trained to treat such corruptions as negligible noise. The same logic, extended to operational fluctuations, accounts for the cross-bridge transfer advantage.

### 6.1. Applicability and Limitations

The framework’s premises define its applicability. The structural-form mismatch between source and target must remain within the augmentation manifold; the bidirectional B3 ↔ Z24 results (with their two- versus three-span asymmetry) mark the practical edge of this envelope. When source and target differ in fundamental structural typology—say, a cable-stayed bridge as the source and a masonry arch as the target—the augmentation manifold cannot bridge the gap, and the irreducible joint risk λ in the transferability bound dominates. In such regimes, multi-source pretraining drawn from a structurally similar population is expected to be more profitable than transfer from a single dissimilar source.

The method also presupposes that the damage modes of interest induce response variations within the frequency band represented in the pretraining data. Damage modes that manifest only in higher-frequency regimes (e.g., minor surface cracking diagnosed via acoustic emission) lie outside the band considered here and would require complementary sensing.

### 6.2. Practical Deployment Considerations

Three operational features ease deployment. First, the pretraining stage requires no labels, so it can be initiated immediately upon installation of an SHM system on the source bridge, without awaiting any damage events. Second, the pretrained encoder is reusable across multiple target bridges within the same structural-form population, amortising the pretraining cost. Third, the fine-tuning stage runs in minutes on modest hardware, making periodic re-calibration practical as additional target-side labelled examples accumulate.

A caveat concerns environmental coverage. If the target bridge experiences operational conditions (temperature range, traffic spectrum) that fall outside those seen by the source during pretraining, the encoder’s invariance to those conditions cannot be guaranteed. Operators should therefore favour source bridges whose monitoring history spans an environmental range encompassing that of the target.

### 6.3. Relationship with Physics-Based Approaches

The proposed framework is complementary to, rather than competitive with, physics-based diagnostic approaches. Finite-element-model-derived features can serve as auxiliary inputs to the few-shot classifier, providing physically interpretable channels alongside the data-driven encoder output. In the inverse direction, the encoder’s t-SNE clusters can be used to validate or refine the damage-class taxonomy assumed by a physics-based model. Hybrid pipelines combining the two are a natural direction for follow-up work.

## 7. Conclusions

Cross-bridge structural damage diagnosis is fundamentally constrained by the asymmetry between abundant unlabelled source-side response and scarce labelled target-side damage instances. This work confronted that bottleneck by coupling a physically grounded siamese contrastive pretraining scheme with a one-dimensional self-calibrated convolutional encoder, enabling environment-invariant representations to be distilled from unlabelled source data and rapidly adapted to a previously unseen bridge using only a handful of labelled samples. On the bidirectional Z24 ↔ laboratory-bridge benchmark, the framework achieved a 5-shot macro-F1 of 0.913 and 0.892 in the two transfer directions, surpassing the strongest of thirteen competing methods—including the state-of-the-art MS-DA and FaultFormer—by 3.4 percentage points and outdistancing training-from-scratch by over 37 points. Component ablations confirmed that the augmentation policy, the self-calibrated encoder, and the SimSiam objective each contribute non-trivially; under additive noise down to 0 dB, the macro-F1 degraded by only 23 percentage points against 30–35 points for the strongest baselines. Beyond demonstrating positive transfer on a single bridge pair, the results indicate that augmentation-driven invariance can substitute principled feature engineering in population-scale SHM. Future work will pursue heterogeneous multi-source pretraining to widen the augmentation manifold, integrate environmental covariates as auxiliary classifier inputs, and develop online incremental fine-tuning, ultimately toward hybrid data–physics pipelines for transportation-network-scale deployment.

## Figures and Tables

**Table 1:** Domain-adapted augmentation policy for cross-bridge contrastive pretraining.

Augmentation	Category	Sampling Distribution	Physical Analogue
Random dropout	Sensor-level	Mask ratio ~ U(0.05, 0.20)	Packet loss, intermittent sensor drop-out
Gaussian noise	Sensor-level	SNR ~ U(15, 30) dB	Electronic noise, ambient interference
Amplitude scaling	Operational	Factor ~ LogU(0.1, 2.0)	Traffic-load variability, thermal modulation of response amplitude
Time-window shifting	Operational	Offset ~ U(−10%, +10%)	Asynchronous observation epochs of the same stationary state
Frequency-band masking	Operational	Band centre ~ U(0, Nyq); width = 5% Nyq; *p* = 0.3	Partial sensor-band failure; cross-bridge resonant-band shift

**Table 2:** Summary of the two datasets used in the experimental evaluation.

Property	Z24 (Open-Source)	Lab Bridge (Self-Built)
Structure type	PT concrete box-girder	Aluminium model bridge
Span arrangement	3 spans (14 + 30 + 14 m)	2–3 spans, four configurations
Sampling frequency	100 Hz	256 Hz (native); resampled to 100 Hz
Sensor count (used)	6	22
Window length	2048 samples (20.48 s)	2048 samples (20.48 s)
Damage states	7 (UD, D1–D7)	10 (UD, M1–M6, SB1–SB3)
Unlabelled pretraining windows	38,400	24,000
Labelled windows per state	up to 200	80–200
Environmental variability	Temperature, humidity (uncontrolled)	Temperature controlled (−15 to +30 °C)

**Table 3:** Principal hyperparameter settings used throughout the experiments.

Stage	Hyperparameter	Value
Pretraining	Optimiser	LARS
Pretraining	Initial learning rate	0.05 (cosine decay)
Pretraining	Batch size	256
Pretraining	Epochs ($T_{1}$)	200
Pretraining	Projection MLP widths	256–256–128
Pretraining	Prediction MLP widths	128–64–128
Encoder	SCConv kernel size	7
Encoder	Downsampling rate r	4
Encoder	Channel widths	64, 128, 256
Fine-tuning	Optimiser	Adam
Fine-tuning	Learning rate (head/encoder)	5 × 10^−4^/5 × 10^−5^
Fine-tuning	Batch size	16
Fine-tuning	Maximum epochs ($T_{2}$)	100
Fine-tuning	Early-stopping patience	15

**Table 4:** Encoder ablation. Macro-F1 at K = 5, Z24 → Lab transfer.

Encoder	Params (M)	Macro-F1	Δ vs. SCConv (pp)
Plain 1D-CNN	0.94	0.846 ± 0.022	−6.7
1D-ResNet-18	1.31	0.872 ± 0.019	−4.1
1D-CNN + SE	1.02	0.879 ± 0.020	−3.4
1D-CNN + CBAM	1.06	0.884 ± 0.019	−2.9
**SCConv (proposed)**	**1.21**	**0.913 ± 0.018**	—

**Table 5:** Pretraining-objective ablation. Macro-F1 at K = 5, Z24 → Lab transfer.

Pretraining Objective	Macro-F1	Δ vs. SimSiam (pp)
No pretraining (supervised from scratch)	0.617 ± 0.041	−29.6
SimCLR	0.871 ± 0.020	−4.2
MoCo v3	0.884 ± 0.019	−2.9
BYOL	0.896 ± 0.019	−1.7
Barlow Twins	0.889 ± 0.020	−2.4
**SimSiam (proposed)**	**0.913 ± 0.018**	—

**Table 6:** Augmentation-component ablation. Macro-F1 at K = 5, Z24 → Lab transfer.

Configuration	Macro-F1	Δ vs. Full (pp)
**Full augmentation policy**	**0.913 ± 0.018**	—
- Dropout	0.901 ± 0.019	−1.2
- Gaussian noise	0.896 ± 0.020	−1.7
- Amplitude scaling	0.878 ± 0.021	−3.5
- Time-window shift	0.887 ± 0.020	−2.6
- Frequency-band masking	0.892 ± 0.020	−2.1
Sensor-level only	0.851 ± 0.022	−6.2
Operational-level only	0.873 ± 0.021	−4.0

**Table 7:** Domain-discrepancy verification (Z24 → Lab B2), measured in the encoder feature space.

Feature Representation	Proxy A-Distance	SWD (a.u.)	Macro-F1 (K = 5)
Raw windowed signal	1.93	4.91	—
Encoder, no pretraining	1.66	3.92	0.617
Encoder, SimCLR pretraining	1.02	1.84	0.871
Encoder, proposed pretraining	0.74	1.31	0.913

**Table 8:** Case study (Lab → Z24, K = 5): per-class detection of the progressive pier-settlement family.

State	Precision	Recall	F1
UD (healthy)	0.96	0.98	0.97
D1 (settlement 20 mm)	0.86	0.83	0.845
D2 (settlement 40 mm)	0.88	0.87	0.875
D3 (settlement 80 mm)	0.93	0.94	0.935

**Table 9:** Computational cost comparison.

Method	Params (M)	FLOPs (G)	Pretraining (h)	Inference (ms/sample)
**Proposed**	1.21	0.38	4.1	1.8
BYOL (1D-CNN)	1.04	0.31	3.6	1.6
FaultFormer	2.84	1.17	9.7	4.3
MS-DA	0.21	0.04	— (no pretraining)	0.4

## Data Availability

The Z24 benchmark data are publicly available from KU Leuven (https://bwk.kuleuven.be/bwm/z24 (accessed on 14 June 2026)). The self-built laboratory dataset and the codes implementing the proposed framework will be made available upon reasonable request to the corresponding author.
